# Dentistry as a Profession

**Published:** 1918-09-07

**Authors:** 


### DENTISTRY AS A PROFESSION.

### Its Drawbacks and Preparations to Qualify.

The dental profession is one that offers many attractions to young men seeking a vocation of real usefulness and profit. Although modern requirements very properly demand from the dentist real medical preparation for his career, and, on the other hand, mechanical training, manipulative skill, and knowledge of such subjects as metallurgy, there is plenty of scope both for those whose tastes are almost entirely mechanical or scientific, and for those to whom either the healing art, or preventive medicine, is more attractive. The relation between general diseases and dental diseases is very close indeed; and conservative dentistry worthy of the name needs, or at any rate is much helped by, a very thorough knowledge of bacteriology. The prosthetic side of the profession—and this is, of course, the most paying branch of dentistry—gives scope for highly developed mechanical knowledge, applied science, inventive ability, and even, in some of its branches, for the artistic faculty. Nor need the dentist do all his own work. He who prefers the surgical and medical side of his work can be assisted by a colleague whose tastes are mechanical; and even a man who dislikes all surgical work can employ skilled and qualified colleagues for this side, while yet using his abilities and applying his scientific knowledge to his cases. Moreover, the dental profession is far from being overcrowded. The demand for qualified dentists far exceeds the supply, and even the unqualified man, whose practice is a public danger, has more than he can do. The young man choosing dentistry as a profession has almost the certainty of a good living in prospect.

One drawback there undoubtedly is, which has done much to prevent many men from entering the profession. It is the very unsatisfactory state of the law in regard to practice by the unqualified. A man naturally hesitates to spend four or five years and a very considerable sum of money in qualifying himself for a dental diploma when he knows that he will have to compete with, and in the eyes of the general public have a status little better than, thousands of advertising quacks. In the present state of the law any man can practise dentistry without any qualification or preparation whatsoever, so long as he does not use the title "Dentist," or append to his name the letters showing his degree or qualification. It is nevertheless possible for the dental profession to do much towards remedying the evil by education of the public, by demanding an amendment of the law, and by so conducting the practice of their profession as to raise it in the eyes of the public and of the medical profession above the level of the tooth-manufacturing companies whose advice and surgery consist, for the most part, in making room in the victim's mouth for the wares they supply. Some fifteen months ago the Lord President of the Council appointed a Departmental Committee to investigate the extent and gravity of the evils connected with the practice of dentistry and dental surgery by persons not qualified under the Dentists Act, to inquire into the expediency of legislation, and to consider and report. The report of this Committee is now awaited.

The first step for the dental student is to register with the General Medical Council, and it is strongly advisable that he should register both as a dental and as a medical student, in case he should wish to complete the curriculum for the conjoint diploma. Four years must elapse between this registration and the final examination for the L.D.S., and although training in dental mechanics and instruction in chemistry and practical chemistry and physics may be done before registration, the time spent at the work on these subjects will not be counted in the four years required unless registration precedes it. The recommendations adopted by the General Medical Council in 1909 as to the course of study and examinations are under revision by the Council. Their consideration has, however, been postponed until the issue of the report of the Departmental Committee referred to above.

The necessary training in dental mechanics may be taken either in the mechanical department of a recognised dental hospital or with a registered practitioner. A Preliminary Science Examination has to be passed in chemistry and physics. The necessary study for this examination also counts as part of the four years of professional study if taken after registration, but not otherwise. This Preliminary Science Examination must be passed previous to the commencement of the two years' hospital course for the final examination. It is therefore advisable, if possible, to take the necessary classes for this purpose and to pass the examination during the first year of study.

When the student has passed his Preliminary Science, has attended lectures on dental mechanics and metallurgy, and completed the manufacture of the necessary dentures and crowns required by the curriculum, he will be in a position to pass the first professional examination. This consists of two parts: I. A practical examination in mechanical dentistry, conducted in the mechanical laboratory of one of the London dental hospitals. II. A written examination in dental metallurgy.

The second, or final, professional examination is in two parts. The first, in medical subjects, must be passed before the second, in dental subjects. It includes general anatomy and physiology, and general pathology and surgery. Part II. consists of dental anatomy and physiology, dental pathology and surgery, and practical dental surgery. These examinations are partly written and partly oral, and the student in offering himself must produce a certificate showing that he has attended lectures on anatomy and physiology, practical physiology, surgery, and medicine at a recognised medical school, and has performed dissections during not

(*Continued on p.* 493.)

### Dentistry as a Profession (*continued from page* 490).

less than twelve months, and attended the practice of surgery and clinical lectures on surgery at a hospital or hospitals for twelve months during ordinary sessions. At the special dental part of the examination the oral examination is conducted by the use of preparations, casts, drawings, etc., and at the practical examination candidates may be examined (*a*) on the treatment of dental caries, on the preparation of teeth by filling with gold or other material, by inlaying or by crowning, and other operations in dental surgery; (*b*) on the mechanical and surgical treatment of the various irregularities of children's teeth. A certificate must have been produced showing that the student has attended at a dental hospital and school: (*a*) a course of dental anatomy and physiology; (*b*) a separate course of dental histology, including the preparation of microscopical sections; (*c*) a course of dental surgery; (*d*) a separate course of practical dental surgery; (*e*) a course of not less than five lectures on the surgery of the mouth which may form part of the course of lectures on surgery required for Part I. of the examination); (*f*) a course of dental bacteriology; (*g*) a course of dental materia medica; (*h*) a course of practical instruction in the administration of such anæsthetics as are in common use in dental surgery. A certificate must also be produced showing that the candidate has, during two years, attended the practice of dental surgery in a dental hospital or the dental department of a general hospital.

The cost of training varies very considerably with the dental schools at which the student enters. One leading school of dentistry charges £150 for the two years' instruction in dental mechanics and the two years' hospital practice and lectures required by the Royal College of Surgeons, with an addition if the fee be paid in instalments. Another very important dental school charges a composition fee of 180 guineas for a two years' course in dental mechanics, chemistry, physics, anatomy, and physiology, and two years' hospital practice, medicine, surgery, and dental lectures, offering registered women students a four years' course, dental curriculum only, for 120 guineas; Besides the London dental hospitals and general hospitals with dental schools, such as Guy's and the London Hospitals and the Royal Free Hospital School of Medicine for Women, which trains its students in conjunction with the two dental hospitals, most of the provincial Universities offer dental training and grant degrees in dentistry.

The Royal Colleges of Surgeons of England, Glasgow, Edinburgh, and Ireland hold examinations for the L.D.S. and grant the diploma.

